# Residual Recurrence of a Small Intestinal Capillary Hemangioma with Obscure Gastrointestinal Bleeding Treated by Double-Balloon Endoscopy: A Case Report and Literature Review

**DOI:** 10.3390/jcm13123415

**Published:** 2024-06-11

**Authors:** Kei Nomura, Tomoyoshi Shibuya, Arisa Yuzawa, Masashi Omori, Rina Odakura, Masao Koma, Kentaro Ito, Eiji Kamba, Takafumi Maruyama, Osamu Nomura, Hirofumi Fukushima, Takashi Murakami, Kumiko Ueda, Dai Ishikawa, Mariko Hojo, Akihito Nagahara

**Affiliations:** Department of Gastroenterology, School of Medicine, Juntendo University, Bunkyo-ku, Tokyo 113-8421, Japan; ke-nomura@juntendo.ac.jp (K.N.); a.nishimura.bp@juntendo.ac.jp (A.Y.); ma-omori@juntendo.ac.jp (M.O.); r.odakura.sp@juntendo.ac.jp (R.O.); m-koma@juntendo.ac.jp (M.K.); k.ito.wj@juntendo.ac.jp (K.I.); e-kamba@juntendo.ac.jp (E.K.); t-maruyama@juntendo.ac.jp (T.M.); onomura@juntendo.ac.jp (O.N.); hfukushi@juntendo.ac.jp (H.F.); t-murakm@juntendo.ac.jp (T.M.); ktamaki@juntendo.ac.jp (K.U.); dai@juntendo.ac.jp (D.I.); mhojo@juntendo.ac.jp (M.H.); nagahara@juntendo.ac.jp (A.N.)

**Keywords:** double-balloon endoscopy, residual recurrence, small bowel capsule endoscopy, small intestinal capillary hemangioma

## Abstract

An 86-year-old man presented with anemia. He underwent abdominal contrast-enhanced computed tomography, gastroscopy, and colonoscopy without any bleeding detected. Small bowel capsule endoscopy (SBCE) revealed a reddish polypoid lesion with blood oozing into the jejunum. Antegrade double-balloon endoscopy (DBE) revealed a 5 mm sized protrusion into the jejunum. Endoscopic mucosal resection (EMR) was difficult; the lesion was snared and resected before energization. Clips prevented further bleeding and the lesion’s position was marked with a tattoo. Histopathological examination of the lesion led to a diagnosis of capillary hemangioma. After 11 months, the patient was again anemic. A reddish polypoid lesion oozing blood near the tattoo was found by SBCE. Another antegrade DBE showed a 7 mm sized protrusion near the tattoo. The lesion was successfully treated by EMR. Histopathological examination revealed the residual recurrence of a small intestinal capillary hemangioma. The patient recovered from anemia after the EMR. Two months later, SBCE showed no findings around the tattoo. Hemangiomas account for 7–10% of benign small intestinal tumors; most are cavernous hemangiomas, and capillary hemangiomas are rare. We report a rare case of a recurring small intestinal capillary hemangioma detected by SBCE and treated using DBE. We also review the literature.

## 1. Introduction

Small intestinal hemangioma is a rare disease, accounting for 7% to 10% of all benign tumors of the small intestine [1,2]. Hemangiomas can be classified as capillary, previously known as pyogenic granuloma, cavernous, or mixed types [3]. Capillary hemangiomas are benign tumors composed of proliferated blood vessels; these predominantly affect the skin but rarely the small intestine. The main presenting symptom of small intestinal capillary hemangiomas is bleeding. Small bowel capsule endoscopy (SBCE) and double-balloon endoscopy (DBE) are also useful in the diagnosis of hemangiomas. In the majority of cases, surgical resections of the small bowel are necessary. However, in recent years, the number of cases treated by DBE have been increasing. There were no reports of residual recurrence of small intestinal hemangiomas. Here, we report a rare case of a residual recurrence of a small intestinal capillary hemangioma that was detected by SBCE and treated using DBE, with a related review of the literature. DBE was suggested as a therapeutic option in this selected case due to its ability to visualize the entire small intestine.

## 2. Case Description

An 86-year-old man presented with anemia at our hospital. He had a medical history of hypertension. Physical examination revealed a height of 174 cm, weight of 64.6 kg, and body mass index of 21.3 kg/m^2^. Vital signs were stable (body temperature was 36.4 °C, blood pressure was 111/79 mmHg, heart rate was 69 bpm, and SpO2 was 98% with room air), and the abdominal examination was unremarkable. Laboratory data showed white blood cells at 7300/µL; hemoglobin at 8.5 g/dL; platelets at 35.8 × 10^4^/µL; blood urea nitrogen at 17 mg/dL; and creatinine at 0.58 mg/dL (Table 1).

**Figure 1:**
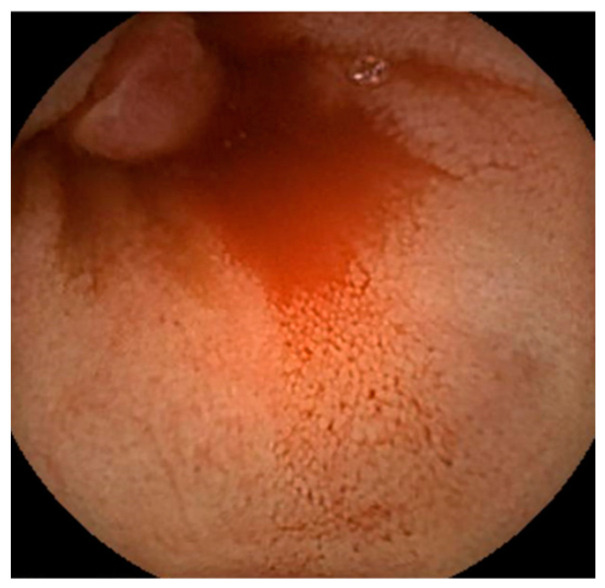
A reddish polypoid lesion and blood oozing into the jejunum as found by small bowel capsule endoscopy.

**Figure 2:**
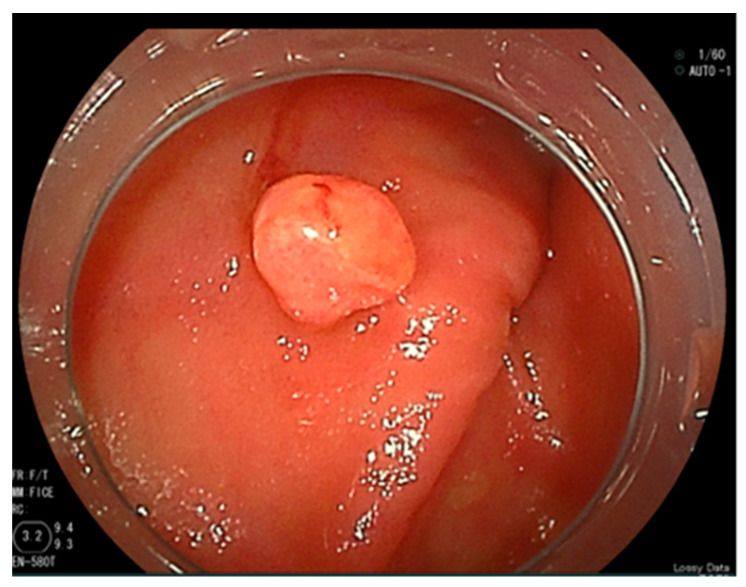
A reddish protrusion in the jejunum as found by double-balloon endoscopy.

Abdominal contrast-enhanced computed tomography, gastroscopy, and colonoscopy were performed; however, no evidence of bleeding was found. An SBCE (PillCam^®^ SB3, Covidien Japan Ltd., Tokyo, Japan) revealed a reddish polypoid lesion with blood oozing into the jejunum (Figure 1). We performed an antegrade DBE (EN-580T^®^, Fujifilm, Tokyo, Japan) to detect the cause of the bleeding and achieved endoscopic hemostasis. The DBE revealed a 5 mm sized protrusion into the jejunum about 200 cm distal from the Treitz ligament (Figure 2). The surface of the lesion was covered with mucus and slightly reddish compared to the surrounding mucosa.

**Figure 3:**
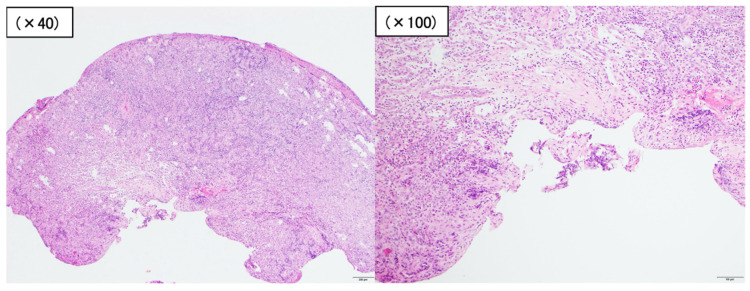
Histological examination of the lesion that was identified as a small intestinal capillary hemangioma with a positive vertical margin (hematoxylin–eosin).

According to these findings, we suspected a non-epithelial tumor such as a hemangioma. Endoscopic mucosal resection (EMR; Snare Master^®^, Olympus, Tokyo, Japan) was attempted, but manipulating the endoscope was difficult, so the lesion was snared and resected before energization. Clips (EZ clip^®^, Olympus, Tokyo, Japan) were used to prevent further bleeding and the position of the lesion was marked with a tattoo. Histological examination of the lesion revealed the dense growth of capillaries; the lesion was therefore diagnosed as a capillary hemangioma (Figure 3). Our follow-up of the patient was uneventful for 11 months and revealed that he had recovered from anemia.

**Figure 4:**
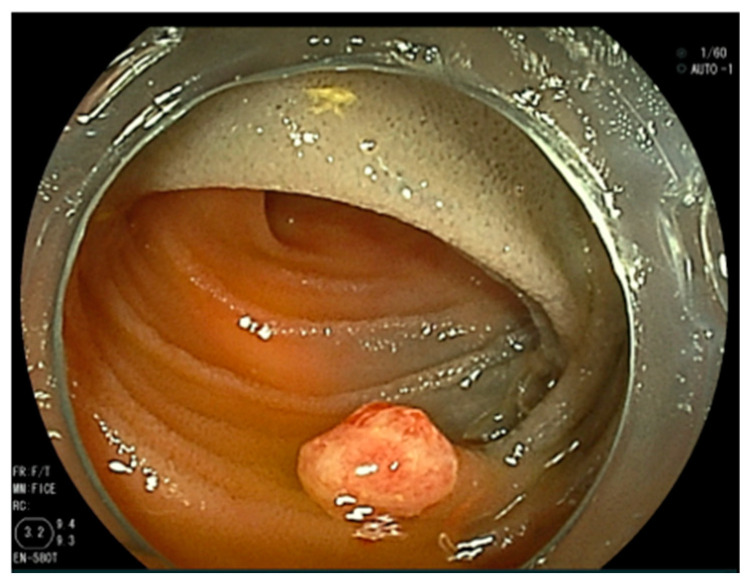
A reddish protrusion near the tattoo in the jejunum as found by double-balloon endoscopy.

**Figure 5:**
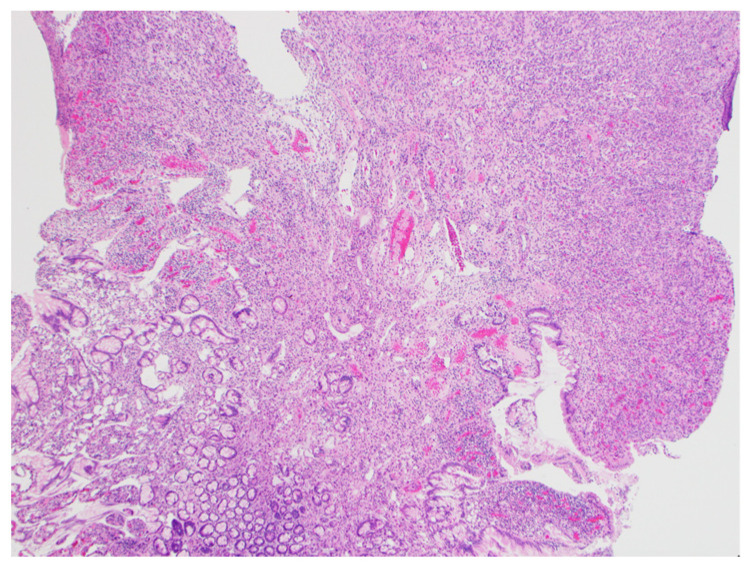
Histological examination of the lesion identified this as a residual recurrence of a small intestinal capillary hemangioma. The vertical margin was negative (hematoxylin–eosin).

After 11 months, however, the patient was found to be anemic again. SBCE revealed a reddish polypoid lesion with blood oozing near the tattoo in the jejunum. DBE was performed again and revealed a 7 mm sized reddish protrusion near the tattoo. The lesion was successfully treated by EMR (Figure 4). Histopathological examination revealed that the bulge was densely populated with capillaries, showing enlarged endothelial cells and rather profuse angiogenesis at the base; the vertical margin was negative (Figure 5). After reviewing the pathology of the previous lesion, the vertical margin was found to be positive. Therefore, the diagnosis was a residual recurrence of a small intestinal capillary hemangioma. The patient recovered from anemia after the EMR. Two months later, an SBCE was performed and no residual small intestinal hemangioma was found.

## 3. Discussion

We here report a rare case of a small intestinal capillary hemangioma that was detected by SBCE and treated using DBE. Also, notably, the small intestinal hemangioma recurred, with the same size, within a short period of time after endoscopic resection. There were no reports of residual recurrence of small intestinal hemangiomas.

The cause of obscure gastrointestinal bleeding is usually difficult to diagnose due to the extensive length of the small intestine. Bleeding from lesions in the small intestine cannot be detected by gastroscopy or colonoscopy [4]. The small bowel is the source of gastrointestinal bleeding in between 2% and 10% of all bleeding cases; the percentage is probably higher among patients experiencing obscure gastrointestinal bleeding [5]. Bleeding in the small intestine is known to occur due to the presence of small intestinal vascular malformation, ulcers, inflammatory bowel disease, or tumors [6]. Vascular tumors of the small bowel are rare, accounting for only between 7% and 10% of all benign tumors in this region [7,8]. Hemangiomas are rare vascular tumors of the gastrointestinal tract. They are an uncommon cause of obscure gastrointestinal bleeding, accounting for between 5% and 10% of all small bowel benign neoplasms [1]. Usually, they occur in the jejunum and can range in size from millimeters to several centimeters for larger forms. They can present as occult or overt obscure gastrointestinal bleeding or complications, such as an obstruction or intussusception [9]. They can be classified as capillary, previously known as pyogenic granuloma, cavernous, or mixed types, according to the size of the injured vessel [3]. Most are cavernous hemangiomas, and capillary hemangiomas are rare. The latter are usually singular, may differ in size, and comprise densely packed submucosal capillaries. Bleeding is the primary symptom they present with. If the hemangiomas are large, they can lead to intussusception and bowel obstruction.

Gastroscopy and colonoscopy have limitations in examinations of the small intestine because of the difficulty in reaching bleeding sites in that region. Therefore, SBCE and DBE have recently been used for diagnosis in cases of small intestinal bleeding [4,10,11]. In patients with obscure gastrointestinal bleeding, capsule endoscopy proves superior to radiography for detecting notable anomalies in the small intestine. Endoscopic findings of gastrointestinal hemangiomas often take the form of a semi-pedunculated to pedunculated submucosal tumor that is blue to dark red in color and easily deforms under pressure. Hemangiomas vary in size, shape, and color. Therefore, it is often difficult to diagnose hemangiomas based on endoscopic findings alone. In our case, SBCE and DBE revealed findings similar to those of a reddish submucosal tumor, suggesting a hemangioma.

In most cases of capillary hemangiomas, surgical resections of the small intestine are performed [12,13,14]. DBE proves to be a valuable method for confirming histology and administering endoscopic treatment of small bowel lesions. EMR using DBE is frequently employed for the treatment of small intestinal polyps [15]. Previously, small intestinal hemangiomas were diagnosed by small bowel enterography or angiography. In most cases, surgical management remains the main treatment for hemangiomas. However, in recent years, the number of cases diagnosed and treated by endoscopic methods, such as SBCE and DBE, have been increasing [16]. The benefit of using DBE for the treatment of a small intestinal hemangioma is that the risk of treatment complications is relatively lower than with surgery. However, potential risks of DBE in treating hemangiomas include bleeding, and intestinal perforation and its consequences. Since intestinal hemangiomas originate from the submucosal layer, endoscopic treatment, such as EMR, has the risk of perforation. Although it is difficult to diagnose a small intestinal hemangioma, once it is diagnosed, enteroscopy may be an effective but non-invasive treatment option. However, if endoscopic manipulation is not easy, prompt surgery is the principal treatment and the indications for surgery should be carefully considered.

We conducted a literature review regarding the endoscopic treatment of small intestinal hemangiomas. We searched medical databases for relevant papers published after 2000, including PubMed and the Japan Medical Abstracts Society, and found 17 prior case reports [17,18,19,20,21,22,23,24,25,26,27,28,29,30,31,32,33] (Table 2). Most of the 32 patients presented with anemia, melena, hematochezia, and gastrointestinal bleeding. The locations were as follows: the jejunum accounted for 17 out of 32 cases (53.1%), and the ileum also accounted for 17 out of 32 cases (53.1%). The size of lesions ranged from 3 mm to 30 mm; endoscopic therapy tended to be used for small lesions. Among them, 4 cases were removed by polypectomy, 14 cases were removed by EMR, 1 case was treated by argon plasma coagulation, and 13 cases were treated by polidocanol injection. Regarding the pathology, it was generally reported that cavernous occurrence is more common; however, most reports of endoscopic treatment of small intestinal hemangiomas showed capillary and pyogenic granulomas. There were no reports of residual recurrence after endoscopic treatment of small intestinal hemangiomas, as occurred in our case.

In this case, we ultimately performed EMR by DBE for the small intestinal hemangioma because the patient was elderly and therefore had a high risk of complications in invasive treatments such as surgery. An EMR of the small intestinal hemangioma may avoid surgery and its attendant risks, and is considered worth trying as a non-invasive treatment. However, when massive bleeding occurs during DBE and endoscopic treatment becomes more difficult, it is necessary to immediately shift to surgery or interventional radiology. Therapeutic interventions using DBE can be performed, even in the deep small bowel, thus avoiding the need for surgery.

As far as we were able to ascertain, all cases recovered after treatment, and no reports were found of residual recurrence of small intestinal hemangiomas. Residual recurrence of small intestinal hemangiomas, as occurred in this case, is rare. Details on the cause of the residual recurrence after endoscopic resection that occurred in this case are unknown because reports on cases of recurrence and the basis for the recurrence are lacking. Regarding colon polyps, after cold snare polypectomy, the frequency of residual recurrence detected at the next colonoscopy was 1.9% (mean follow-up period: 13.0 ± 4.0 months) [34]. Higher rates exist for recurrent lesions regarding a histopathological positive margin. In this case, the possibility that some hemangiomas remained after the initial resection cannot be ruled out. Therefore, to prevent recurrence, it is desirable to perform endoscopic resection without leaving any tumor behind. Additionally, it was reported that hemangiomas infiltrated the deeper serosal layer through the connective tissue within the muscle layers. Therefore, it is advisable to undergo preoperative endoscopic ultrasonography (EUS) to prevent incomplete endoscopic resection [35]. In this case, it was difficult to perform EUS adequately because of the poor manipulation of DBE. Therefore, we performed EMR without EUS. However, as mentioned above, the small intestinal wall is thin and a risk of perforation exists. Furthermore, since it may be difficult to completely remove endoscopically a hemangioma that shows deep invasion, a combination of endoscopic treatment and surgery should be considered depending on the morphology of the tumor.

## 4. Conclusions

In summary, we report that the residual recurrence of a small intestinal capillary hemangioma with obscure gastrointestinal bleeding was treated by EMR using DBE, thereby avoiding surgery and its attendant risks. This treatment may be a potential non-invasive option for a small intestinal capillary hemangioma. It is recommended that careful consideration be given to any indications for endoscopic treatment because endoscopic intervention may lead to uncontrolled bleeding or perforation. The cause of the rapid recurrence of the small intestinal hemangioma is unknown and further research is needed.

## Figures and Tables

**Table 1:** Laboratory findings on admission.

White blood cells	(/µL)	7300
Red blood cells	(/µL)	322 × 10^4^
Hemoglobin	(g/dL)	8.5
Hematocrit	(%)	27.5
Mean corpuscular volume	(fL)	85.4
Reticulocytes	(%)	1.2
Platelets	(/µL)	35.8 × 10^4^
Aspartate aminotransferase	(U/L)	15
Alanine aminotransferase	(U/L)	15
Lactate dehydrogenase	(U/L)	141
Alkaline phosphatase	(U/L)	133
Total protein	(g/dL)	7.7
Albumin	(g/dL)	3.4
Blood urea nitrogen	(mg/dL)	17
Creatinine	(mg/dL)	0.58
Sodium	(mEq/L)	137
Potassium	(mEq/L)	4.7
Chloride	(mEq/L)	102
Total Bilirubin	(mg/dL)	0.45
Iron	(µg/dL)	35
Total iron-binding capacity	(µg/dL)	338
Unsaturated iron-binding capacity	(µg/dL)	303
Ferritin	(ng/dL)	16
C-reactive protein	(mg/dL)	2.14
Carcinoembryonic antigen	(ng/mL)	1.7
Carbohydrate antigen 19-9	(U/mL)	5

**Table 2:** Review of literature on endoscopic treatment of small intestinal hemangiomas.

Reference	Age	Sex	Complaint	Diagnosis	Location	Single/ Multiple	Size	Treatment	Pathology
Yamamoto [17]	57	F	Melena	CS	Terminal ileum	Single	7 mm	Polypectomy	Capillary
Shirakawa [18]	72	M	Gastrointestinal bleeding	SBCE, DBE	Jejunum	Single	-	Polypectomy	Pyogenic granuloma
Willert [19]	19	M	Anemia	SBCE, DBE	Jejunum/ileum	Multiple	8 mm/ 14 mm	EMR	Cavernous
Ng [20]	20	F	Anemia	Small bowel enema, CS	Terminal ileum	Multiple	-	APC	-
Kuga [21]	55	M	Anemia	SBCE, DBE	Jejunum	Single	4 mm	Polypectomy	Pyogenic granuloma
Shibuya [22]	74	M	Melena	SBCE, DBE	Jejunum	Single	19 mm	EMR	Capillary
Nagoya [23]	63	F	Anemia	SBCE, DBE	Ileum	Single	7 mm	EMR	Pyogenic granuloma
Easler [24]	71	M	Anemia and melena	BAE	Jejunum	Single	-	EMR	Cavernous
Mizusawa [25]	73	M	Anemia	SBCE, DBE	Jejunum	Single	6 mm	EMR	Capillary
Hirata [26]	82	F	Gastrointestinal bleeding	SBCE, DBE	Ileum	Single	8 mm	EMR	Pyogenic granuloma
Ning [27]	10	M	Melena	BAE	Jejunum/ileum	Multiple	3–30 mm	Polidocanol injection	-
Kawasaki [28]	86	F	Gastrointestinal bleeding	SBCE, DBE	Ileum	Single	7 mm	EMR	Pyogenic granuloma
Mizutani [29]	58	F	Melena and anemia	SBCE, DBE	Jejunum	Single	-	EMR	Pyogenic granuloma
Igawa [30]	-	6M/6F	Gastrointestinal bleeding	SBCE, DBE	Eight jejunum/four ileum	Seven single/ five multiple	-	Polidocanol injection	-
Romero [31]	46	F	Anemia	SBCE, BAE	Jejunum	Single	15 mm	EMR	Pyogenic granuloma
Zhang [32]	67	M	Abdominal pain	CS	Ileum	Single	8 mm	Polypectomy	Pyogenic granuloma
Hayashi [33]	53–82	2M/3F	Hematochezia and anemia	SBCE, BAE	Ileum	Four single/ one multiple	4–7 mm	EMR	Pyogenic granuloma
Present case	86	M	Anemia	SBCE, DBE	Jejunum	Single	5 mm	CSP	Capillary
Present case	87	M	Anemia	SBCE, DBE	Jejunum	Single	7 mm	EMR	Capillary

APC: argon plasma coagulation, BAE: balloon-assisted enteroscopy, CS: colonoscopy, CSP: cold snare polypectomy, DBE: double-balloon endoscopy, EMR: endoscopic mucosal resection, and SBCE: small bowel capsule endoscopy.

## Data Availability

The data are available upon reasonable request. The data are not publicly available due to patient privacy.

## References

[1] Ramanujam P.S., Venkatesh K.S., Bettinger L., Hayashi J.T., Rothman M.C., Fietz M.J. (1995). Hemangioma of the small intestine: Case report and literature review. Am. J. Gastroenterol..

[2] Wei Y., Yi N., Mo Y., Liang Y. (2023). A case report of gastrointestinal hemorrhage cause by small intestinal hemangioma. Medicine.

[3] Camilleri M., Chadwick V.S., Hodgson H.J. (1984). Vascular anomalies of the gastrointestinal tract. Hepatogastroenterology.

[4] Omiya N., Nakagawa Y., Nagasaka M., Tahara T., Shibata T., Nakamura M., Hirooka Y., Goto H., Hirata I. (2015). Obscure gastrointestinal bleeding: Diagnosis and treatment. Dig. Endosc..

[5] Ell C., May A. (2006). Mid-gastrointestinal bleeding: Capsule endoscopy and push-and-pull enteroscopy give rise to a new medical term. Endoscopy.

[6] He Q., Bai Y., Zhi F.C., Gong W., Gu H.X., Xu Z.M., Cai J.Q., Pan D.S., Jiang B. (2013). Double-balloon enteroscopy for mesenchymal tumors of small bowel: Nine years’ experience. World J. Gastroenterol..

[7] Boyle L., Lack E.E. (1993). Solitary cavernous hemangioma of small intestine. Case report and literature review. Arch. Pathol. Lab. Med..

[8] Garvin P.J., Herrmann V., Kaminski D.L., Willman V.L. (1979). Benign and malignant tumors of the small intestine. Curr. Probl. Cancer.

[9] Morgan D.R., Mylankal K., el Barghouti N., Dixon M.F. (2000). Small bowel haemangioma with local lymph node involvement presenting as intussusception. J. Clin. Pathol..

[10] Otani K., Watanabe T., Shimada S., Hosomi S., Nagami Y., Tanaka F., Kamata N., Taira K., Yamagami H., Tanigawa T. (2018). Clinical utility of capsule endoscopy and double-balloon enteroscopy in the management of obscure gastrointestinal bleeding. Digestion.

[11] Mönkemüller K., Neumann H., Meyer F., Kuhn R., Malfertheiner P., Fry L.C. (2009). A retrospective analysis of emergency double-balloon enteroscopy for small-bowel bleeding. Endoscopy.

[12] Wardi J., Shahmurov M., Czerniak A., Avni Y. (2007). Clinical challenges and images in GI. Capillary hemangioma of small intestine. Gastroenterology.

[13] Kavin H., Berman J., Martin T.L., Feldman A., Forsey-Koukol K. (2006). Successful wireless capsule endoscopy for a 2.5-year-old child: Obscure gastrointestinal bleeding from mixed, juvenile, capillary hemangioma-angiomatosis of the jejunum. Pediatrics.

[14] Kim Y.S., Chun H.J., Jeen Y.T., Um S.H., Kim C.D., Hyun J.H. (2004). Small bowel capillary hemangioma. Gastrointest. Endosc..

[15] May A., Nachbar L., Pohl J., Ell C. (2007). Endoscopic interventions in the small bowel using double balloon enteroscopy: Feasibility and limitations. Am. J. Gastroenterol..

[16] Hu P.F., Chen H., Wang X.H., Wang W.J., Su N., Shi B. (2018). Small intestinal hemangioma: Endoscopic or surgical intervention? A case report and review of literature. World J. Gastrointest. Oncol..

[17] Yamamoto N., Hoshihara Y., Shida K., Hoteya S., Iizuka T., Tanaka Y., Hashimoto M., Yamamoto T., Ubara Y., Tanimoto A. (2004). A case of capillary hemangioma of the ileum diagnosed by endoscopic polypectomy. Gastroenterol. Endosc..

[18] Shirakawa K., Nakamura T., Endo M., Suzuki K., Fujimori T., Terano A. (2007). Pyogenic granuloma of the small intestine. Gastroenterol. Endosc..

[19] Willert R.P., Chong A.K. (2008). Multiple cavernous hemangiomas with iron deficiency anemia successfully treated with double balloon enteroscopy. Gastrointest. Endosc..

[20] Ng E.K., Cheung F.K., Chiu P.W. (2009). Blue rubber bleb nevus syndrome: Treatment of multiple gastrointestinal hemangiomas with argon plasma coagulator. Dig. Endosc..

[21] Kuga R., Furuya C.K., Fylyk S.N., Sakai P. (2009). Solitary pyogenic granuloma of the small bowel as the cause of obscure gastrointestinal bleeding. Endoscopy.

[22] Shibuya T., Osada T., Mitomi H., Takeda T., Nomura O., Nakayama H., Hidaka Y., Mori H., Beppu K., Sakamoto N. (2010). Jejunal capillary hemangioma treated by using double-balloon endoscopy (with video). Gastrointest. Endosc..

[23] Nagoya H., Tanaka S., Tatsuguchi A., Mitsui K., Ehara A., Kobayashi T., Fujimori S., Sakamoto C. (2010). Rare cause of obscure gastrointestinal bleeding due to pyogenic granuloma in the ileum detected by capsule endoscopy and treated with double balloon endoscopy. Dig. Endosc..

[24] Easler J.J., Papachristou G.I. (2012). A case of obscure gastrointestinal bleeding. Gastroenterology.

[25] Mizusawa Y., Shibuya T., Osada T., Kurashita E., Miura M., Nomura O., Fukuo Y., Beppu K., Sakamoto N., Nagahara A. (2013). Two cases of capillary hemangioma of the small intestine treated using double-balloon endoscopy. Prog. Dig. Endosc..

[26] Hirata K., Hosoe N., Imaeda H., Naganuma M., Murata H., Ueno M., Suzuki H., Ogata H., Kanai T. (2014). Obscure gastrointestinal bleeding: Resection of a pyogenic granuloma of the ileum via double-balloon enteroscopy. Clin. J. Gastroenterol..

[27] Ning S., Zhang Y., Zu Z., Mao X., Mao G. (2015). Enteroscopic sclerotherapy in blue rubber bleb nevus syndrome. Pak. J. Med. Sci..

[28] Kawasaki K., Kurahara K., Matsumoto T. (2015). Pyogenic granuloma of the ileum depicted by small-bowel radiography, capsule endoscopy and double balloon endoscopy. Dig. Liver Dis..

[29] Mizutani Y., Hirooka Y., Watanabe O., Nakamura M., Yamamura T., Ando T., Goto H. (2015). Pyogenic granuloma of the small bowel treated by double-balloon enteroscopy. Gastrointest. Endosc..

[30] Igawa A., Oka S., Tanaka S., Kunihara S., Nakano M., Chayama K. (2016). Polidocanol injection therapy for small-bowel hemangioma by using double-balloon endoscopy. Gastrointest. Endosc..

[31] Romero-Mascarell C., García-Pagán J.C., Araujo I.K., Llach J., González-Suárez B. (2016). Pyogenic granuloma in the jejunum successfully removed by single-balloon enteroscopy. Rev. Esp. Enferm. Dig..

[32] Zhang D., Glover S.C., Liu W., Liu X., Lai J. (2019). Small Bowel Pyogenic Granuloma with Cytomegalovirus Infection in a Patient with Crohn’s Disease (Report of a Case and Review of the Literature). In Vivo.

[33] Hayashi Y., Hosoe N., Takabayashi K., Kamiya J.K., Mutaguchi M., Miyanaga R., Hirata K., Fukuhara S., Mikami Y., Sujino T. (2020). Clinical and Endoscopic Characteristics of Pyogenic Granuloma in the Small Intestine: A Case Series with Literature Review. Intern. Med..

[34] Murakami T., Yoshida N., Yasuda R., Hirose R., Inoue K., Dohi O., Kamada K., Uchiyama K., Konishi H., Naito Y. (2020). Local recurrence and its risk factors after cold snare polypectomy of colorectal polyps. Surg. Endosc..

[35] Motohashi Y., Hisamatsu T., Ikezawa T., Matsuoka S., Ogawa S., Yoshino K., Koide O., Mizuno Y., Nishida J. (1999). A case of pyogenic granuloma in the small intestine. Nihon Shokakibyo Gakkai Zasshi.

